# Prediction of arrhythmogenesis in non-ischemic cardiac resynchronization therapy patients

**DOI:** 10.1063/5.0337754

**Published:** 2026-07-14

**Authors:** Alessandra Corda, Massimiliano Maines, Stefano Pagani, Domenico Catanzariti, Maurizio Del Greco, Christian Vergara

**Affiliations:** 1 LaBS, Dipartimento di Chimica, Materiali e Ingegneria Chimica “Giulio Natta”, Politecnico di Milano, Milano, Italy; 2 Cardiology Department, S.M. del Carmine Hospital, APSS Corso Verona, 4, Rovereto 38068, Trento, Italy; 3 MOX, Dipartimento di Matematica, Politecnico di Milano, Milano, Italy

## Abstract

We aim to develop a patient-specific computational model to predict the risk of Ventricular Tachycardia (VT) in patients with a Biventricular Cardiac Resynchronization Therapy (BiV-CRT) device. Patients are indeed at risk of developing arrhythmias due to BiV-CRT pacing, a known potential complication that requires preventive evaluation. We consider three non-ischemic fibrotic patients. Patient-specific left ventricle geometries and fibrosis are extracted from Cine-MRI and LGE-MRI. The electrophysiology model, based on the monodomain equation and the Ten Tusscher–Panfilov (TTP06) ionic model, is personalized using pre-operative Electro-Anatomical Mapping System data. The TTP06 parameters are adapted to reflect the altered electrical properties of the fibrotic tissue. To test inducibility, we use an S1–S2 stimulation protocol: S1 simulates the clinical BiV-CRT pacing with a patient-specific VV-delay, followed by an S2 ectopic impulse. This procedure is repeated for 10 ectopic sites. The arrhythmogenic risk is quantified by the number of ectopic sites that generate a reentry loop. The model's prediction of VT risk in our simulations matches the clinical outcome for all three patients, recognizing the two with spontaneous VT episodes and the one non-arrhythmic patient. Arrhythmic patients show a higher number of ectopic sites from which a reentry loop is generated compared to the non-arrhythmic patient. This study provides a preliminary attempt toward the use of computational tools to assess the vulnerability of the arrhythmic substrate during BiV-CRT pacing in non-ischemic patients. In the future, such tools could serve as a noninvasive diagnostic metric to inform clinicians about possible therapies to be associated with BiV-CRT.

## I. INTRODUCTION

*Biventricular Cardiac Resynchronization Therapy* (BiV-CRT) represents a clinical standard of care for patients suffering from ventricular dyssynchrony, a condition that impairs the synchronism of the cardiac contraction causing heart failure. By restoring regular ventricular motion, BiV-CRT aims to improve systolic function and cardiac output.^1^ Despite significant advances in the pacing field, such as Conduction System Pacing, traditional BiV-CRT remains one of the preferred first-line treatments for cardiac resynchronization.^2^

However, BiV-CRT carries important risks that can lead to adverse outcomes, such as arrhythmogenesis.^3,4^ Clinical studies on BiV-CRT populations have reported a prevalence of non-sustained Ventricular Tachycardia (VT) in approximately 13% of patients, while sustained episodes occur in about 1% of cases.^5^ Indeed, BiV-CRT may introduce electrical instabilities due to its intrinsic non-physiological activation. This induces a reversal of the physiological endocardial-to-epicardial depolarization direction to an epicardial-to-endocardial direction, which is hypothesized to promote heterogeneities during repolarization. This could prolong the QT interval and consequently result in malignant arrhythmias.^6,7^ Such a risk is further increased in BiV-CRT patients by the presence of ischemic or non-ischemic fibrosis.^8^

Given these risks, it would be clinically important to develop reliable methods to identify which patients included in the BiV-CRT program could face an elevated arrhythmic propensity after implantation. In this way, clinicians could assess whether it would be advisable to implant a *CRT Defibrillator* (CRT-D) device.^2^

Computational cardiac models are able to produce the action potential along the whole heartbeat in virtual, clinically relevant, scenarios for a patient.^9,10^ Specifically, over the last decade, computational research has focused on patient-specific models, particularly for individuals with myocardial fibrosis.^11^ This has allowed to develop several computational studies with the aim of analyzing or improving BiV-CRT: evaluating acute hemodynamic changes associated with stimulation;^12^ identifying non-responders through detailed electro-mechanical simulations;^13,14^ optimizing electrode placement and VV-delay;^15,16^ and evaluating unidirectional block formation when the electrode is located near a scarred region.^17^ Moreover, some computational studies have recently focused on the prediction of arrhythmias in several scenarios, such as acute ischemia,^18–20^ chronic ischemia,^21,22^ and non-ischemic fibrosis.^23^

In this study, we present an original computational framework in which a personalized BiV-CRT model is applied to patients with non-ischemic fibrosis to assess the validity of the computational prediction of VT during BiV-CRT. While components such as CRT modeling^12–17^ or fibrosis-related VT inducibility^23^ have so far been studied separately in the literature, this work proposes a comprehensive integration of patient-specific activation data, localized ionic remodeling for non-ischemic fibrosis, and CRT-specific pacing protocols to predict VT specifically in the BiV-CRT clinical population. To this aim, for each patient we personalize the electrical properties of the computational model using activation times obtained from an Electro-Anatomical Mapping System (EAMS),^24,25^ and we propose an ionic current model for non-ischemic myocardial fibrosis regions. Then, we run electrophysiology simulations employing an S1–S2 stimulation protocol adapted to BiV-CRT cases, to assess the patient's propensity for VT formation. To validate our outcomes, we compare the predicted VT risk with the clinical follow-up available at the patient's disposal.

## II. RESULTS

The results of the patient-specific electrophysiological simulations are presented here, structured in two sections: first, we analyze the resulting electrical activation pattern of the left ventricle under standard BiV-CRT pacing (Sec. II A); second, we detail the arrhythmogenic risk assessment derived from the virtual ectopic stimulation protocol (Sec. II B).

### A. Activation pattern under BiV-CRT pacing

**FIG. 1.**
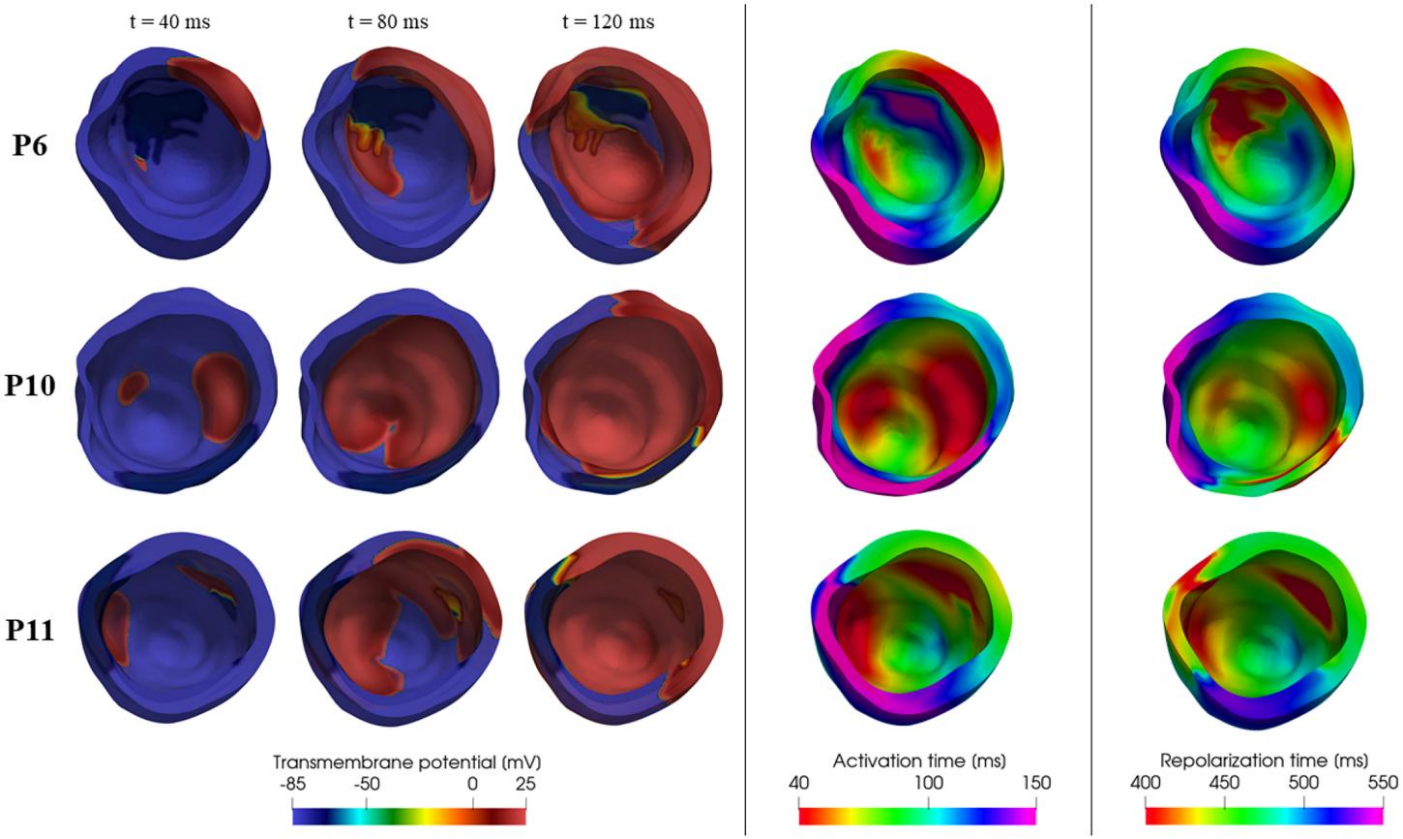
Electrophysiological maps for patients P6, P10, and P11 (rows) under BiV-CRT pacing. Left columns: transmembrane potential propagation at t=40,80, and 120 ms. Center column: activation time maps. Right column: repolarization time maps.

Numerical simulations of the personalized models are run under the BiV-CRT virtual pacing protocol (Sec. V E). In this first analysis, we simulate the post-operative scenarios without ectopic beats to assess the resulting electrical pattern under therapeutic pacing. These simulations incorporate all patient-specific model components, including the personalized monodomain conductivities (Table I) and the adapted TTP06 model used to characterize the electrical behavior of the fibrotic tissue (Sec. V D). Figure 1 shows the propagation of the electrical impulses activated by BiV-CRT with the respective activation and repolarization maps.

**TABLE I.** Top: Values of the personalized conductivity along the fibers ( $\sigma_{f}$), sheets ( $\sigma_{s}$), and normal direction ( $\sigma_{n}$), normalized with respect to the surface-to-volume ratio and the transmembrane capacitance. Values are in ( $10^{-4}\;m^{2}/\text{ms}$). Bottom: Conduction Velocities (CVs) along the three directions estimated from the propagation. Values are in ( cm/s). H stands for healthy region; F stands for fibrotic region.

Patient	$\sigma_{f,H}$	$\sigma_{f,F}$	$\sigma_{s,H}$	$\sigma_{s,F}$	$\sigma_{n,H}$	$\sigma_{n,F}$
P6	1.75	1.40	0.28	0.24	0.07	0.05
P10	1.42	1.02	0.35	0.25	0.08	0.06
P11	1.42	0.60	0.37	0.15	0.08	0.03

	$CV_{f,H}$	$CV_{f,F}$	$CV_{s,H}$	$CV_{s,F}$	$CV_{n,H}$	$CV_{n,F}$
P6	81.6	58.3	31.4	30.3	15.3	13.8
P10	73.5	60.9	36.6	31.2	17.5	15.1
P11	72.8	48.4	37.2	24.2	17.3	10.7

We notice that our simulations are able to reproduce the double pacing at the apex and at the epicardial veins characteristic of BiV-CRT. Even though this was not directly validated against BiV-CRT EAMS measurements (not available), as discussed in Sec. V C, our computational model was validated in Ref. 16 against EAMS measurements in the case of right pacing. Therefore, we can assume that also in the case of biventricular pacing, our model achieves a high level of accuracy and prediction.

### B. Arrhythmogenic risk assessment

**FIG. 2.**
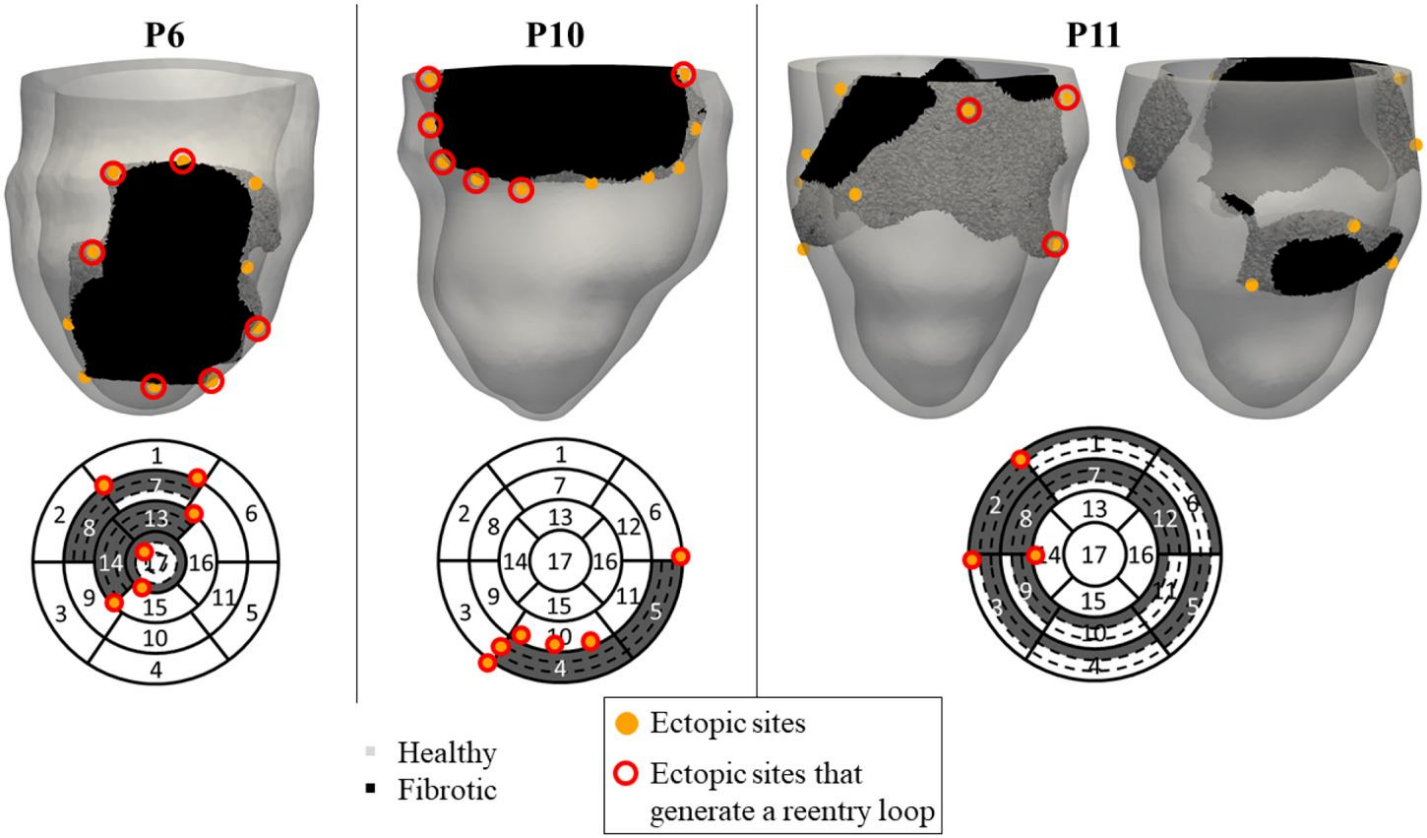
Localization of the 10 ectopic sites across the three patients. The ventricular geometries illustrate all 10 ectopic sites (orange circles). Sites that generate a reentry loop are highlighted with a red ring. The corresponding bullseye plots show only the ectopic sites that are arrhythmogenic.

The personalized models are then subjected to the virtual stimulation protocol described in Sec. V E, where a total of 10 distinct ectopic impulses are applied around the fibrotic region for each patient (see Fig. 2, orange bullets). The corresponding CI ranges between 420 and 550 ms. In the same figure, we depict in red those ectopic impulses that generate a reentry loop. The results regarding the arrhythmogenic risk of each patient together with the clinical follow-up indications are summarized in Table II.

**TABLE II.** Results of the arrhythmogenic risk assessment. The second column reports the number of ectopic sites (out of ten) that resulted in the formation of a reentry loop. The third column reports the documented clinical status.

Patient	Arrhythmogenic ectopic sites/10	Clinical follow-up
P6	6	Arrhythmic
P10	6	Arrhythmic
P11	3	Non-arrhythmic

**FIG. 3.**
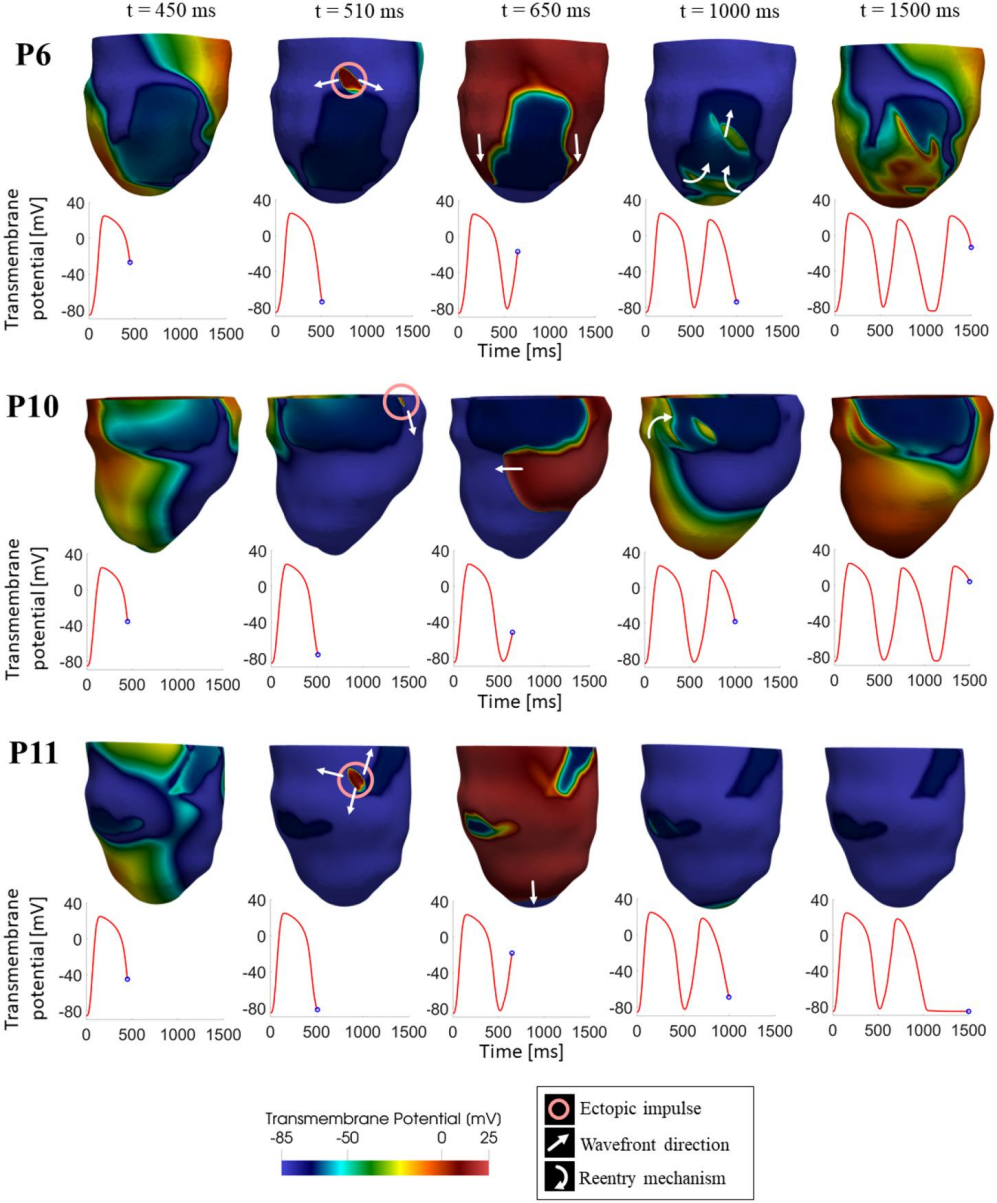
Transmembrane potential propagation during BiV-CRT pacing and subsequent ectopic impulse, shown at five time instants. In P6 and P11, the ectopic beat initiates a reentry, visible as a third activation spike in the plots. In P11, the ectopic impulse decays without triggering reentry.

The computational study shows that the number of ectopic impulses that generate a reentry loop is higher for patients P6 and P10 (6 and 6 out of 10, respectively) compared to that for patient P11 (3 out of 10). This observed pattern is consistent with the documented clinical status of patients P6 and P10 as arrhythmic, and patient P11 as non-arrhythmic (Tables II and III). Figure 3 provides a visual example of a successful reentry loop initiated by an ectopic stimulus in P6 and P10, along with an example of a decaying ectopic impulse in P11. In all cases of induced arrhythmia, the reentrant activity eventually self-extinguishes within the simulated timeframe. While no degeneration into ventricular fibrillation is observed, the initiation of these organized loops serves as a critical indicator of a vulnerable substrate and an increased risk of reentrant VT.

**TABLE III.** Values of delay adjusted by clinicians (VV-delay) and clinical follow-up for each patient. Acronyms: Ventricular Tachycardia (VT); Antitachycardia Pacing (ATP).

Patient	VV-delay (ms)	Follow-up
P6	30	Unsustained VT (6 s)
P10	20	VT terminated with ATP and shock
P11	0	No events

To assess the impact of intramural properties on the VT trigger threshold, we perform a sensitivity analysis on patients P6 and P11 by incorporating transmural electrophysiological heterogeneity (modeling the transmurality in 40% endocardium, 40% myocardium, and 20% epicardium^26^). Specifically, the TTP06 parameters previously used in this study are assigned to the mid-myocardial layer, while the endocardium and epicardium are modeled using their respective electrophysiological properties. New computational meshes are generated and the simulations are re-run. For P6, the number of arrhythmogenic sites is 5/10 (compared to 6/10 in the baseline model), while for P11, no reentry loops are observed (0/10 compared to 3/10). These findings suggest that while transmural gradients can refine the inducibility threshold, the patient-specific arrhythmogenic risk remains primarily dictated by the fibrotic substrate distribution.

## III. DISCUSSION

The present study demonstrated a novel application of a patient-specific computational cardiac model to assess the risk of VT in patients undergoing BiV-CRT who also suffer from non-ischemic myocardial fibrosis.

**FIG. 4.**
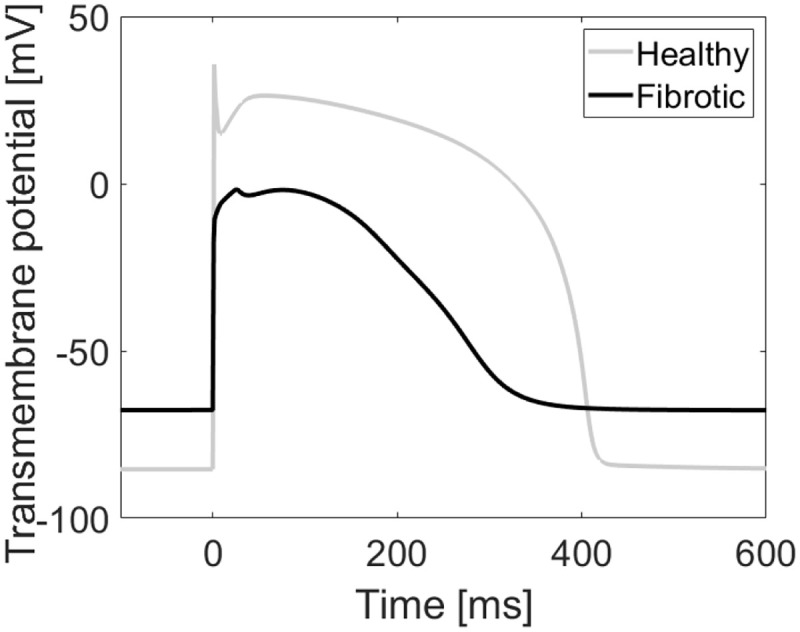
Action potential (AP) morphologies for healthy (gray) and fibrotic (black) regions. The fibrotic AP is obtained by altering specific TTP06 model parameters as in Table IV.

The personalized computational setup, which incorporated patient-specific monodomain conductivities (Table I) and an adapted Ten Tusscher–Panfilov (TTP06) ionic current model for non-ischemic fibrosis (Sec. V D), reliably reflected the individual conduction properties affected by the pathology. This approach was essential for capturing the altered electrical behavior within the arrhythmogenic substrate (Fig. 4).

It is well established that myocardial fibrosis is a complex, heterogeneous substrate that creates surviving myocardial bundles, which may be primary drivers of reentrant arrhythmias.^28^ While some computational studies incorporated these conduction channels by manually designing them,^29,30^ we are not aware of any studies of this type that include a patient-specific design of the channels within the fibrosis. Accordingly, in computational studies where patient-specific reconstruction of fibrosis was considered, they were all accompanied by the modeling assumption of the absence of conduction in such regions.^12,31^ Since our primary goal was to develop a predictive tool relying exclusively on data acquired during the standard clinical BiV-CRT procedure (LGE-MRI and, possibly, EAMS) and since specific, nonstandard acquisitions would be needed to detect patient-specific fibrotic channels (such as Ultra High-Density mapping), we tried to overcome the limitation introduced by other computational studies by using the action potential reported in Fig. 4 for the cells of the fibrosis. This AP should then be thought as an “average” value among the fibrotic cells, which, although did not allow us to identify the channels, better characterized the electrophysiology in fibrosis compared to considering all its cells as non-conducting.

The BiV-CRT simulations in the absence of any ectopic beat allowed us to characterize the typical electrical propagation patterns under this pacing condition. These results confirmed the expected double activation wave initiated at the apex and the epicardial veins, demonstrating the model's capability to accurately simulate the post-operative electrical state under pacing conditions (Fig. 1). Specifically, the activation maps revealed heterogeneous conduction among the three patients, consistent with their personalized structural and electrical properties. Moreover, the repolarization maps highlighted significant differences in the distribution and dispersion of the repolarization time, indicating patient-specific electrophysiological vulnerabilities even under therapeutic pacing conditions.

As regards the fiber disposition inside the myocardial wall, notice that our choice of values of (−60, +60) degrees is widely considered in several studies as a reliable representation of human cardiac fibers.^32–35^ Nonetheless, we account for the fact that this property depends largely on the individual, and therefore our choice is only one of many possible options.^36^ In order to address how much this choice influences our outcomes, we performed for patient P6 the simulations for all the ectopic cases also for the choice of values of (−70, +80). We found that the results showed no significant changes in terms of activation patterns, arrhythmic dynamics, and number of reentries.

The primary finding of this study was the predictive coherence between the computationally assessed VT risk obtained by introducing ectopic beats during BiV-CRT pacing and the long-term clinical follow-up of BiV-CRT patients with non-ischemic fibrosis (Table II). Patients P6 and P10, who were clinically classified as arrhythmic, showed a significantly higher arrhythmogenic propensity, with 6 ectopic impulses out of 10 that formed a reentry loop, compared to the non-arrhythmic patient P11 (who had only 3 ectopic sites with loop formation).

It is interesting to notice the figure-of-eight morphology of the reentrant loops (Fig. 3). This structure is typically observed in VT events, which are characterized by organized circuits often linked to the presence of an anatomical obstacle like scars.^37^ By successfully simulating VT, our model showed clear coherence with the clinical follow-up of the studied patients, in which chaotic events such as Ventricular Fibrillation (VF) were not observed. The initiation of these organized loops consistently occurred around the fibrotic region, confirming that changes in conductivity and altered cellular properties modified the substrate to induce an increased refractory period and subsequent organized reentry. Specifically, the alterations of the ionic parameters and the 3D conductivities inside the fibrotic regions led to a higher Effective Refractory Period (ERP) and to conduction slowing, respectively. We believe that the interplay between these two factors is the main factor responsible for unidirectional blocks and consequent reentry loop formation. However, we did not investigate more deeply which of the two might play a major role, and we acknowledge that our study does not isolate their respective contributions.

Focusing specifically on the role of ERP, we may recognize, in general, two opposite behaviors: (i) a tendency toward shortening due to the shortening of APD;^38^ (ii) a tendency toward prolongation due to the elevated resting potential, which is caused by a slowdown of the sodium channel recovery.^39^ In our case, it could be hypothesized that in the fibrosis the ERP is larger than in the healthy tissue, since, by looking at Fig. 3, it seems that the propagating front of the ectopic impulse encounters a unidirectional block, probably due to refractoriness, from which the loop originates. However, we are aware of the fact that our analysis cannot provide a definitive answer regarding the role of ERP in arrhythmogenesis.

**FIG. 5.**
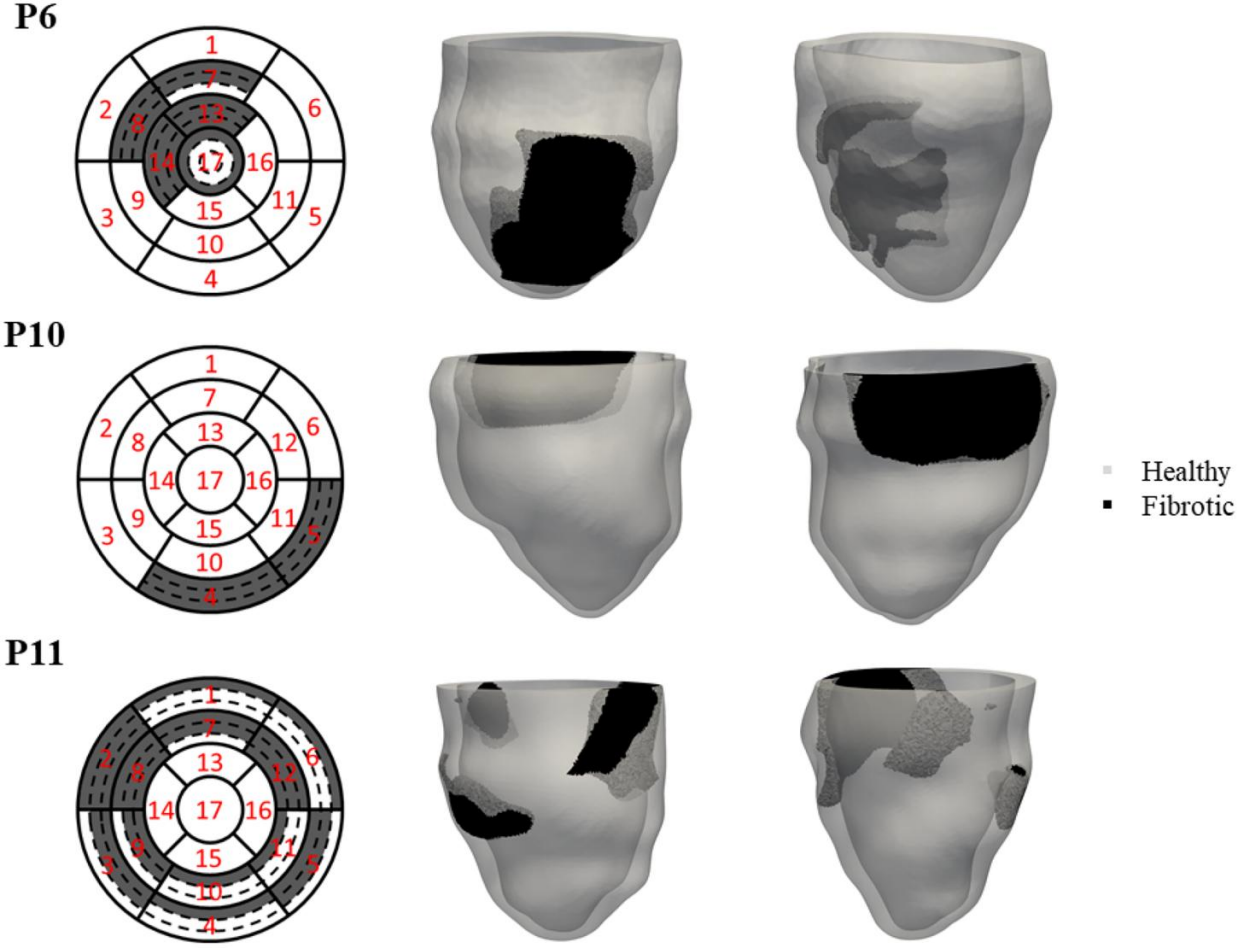
3D reconstruction of the LV and fibrosis for patients P6, P10, and P11. Fibrotic regions are derived from the clinicians' bullseye plots, which are adapted to represent the endocardial, myocardial, and epicardial layers within the affected segments.

Although the number of cases is very limited, we try here to provide a possible rationale behind the persistent formation of reentries. From the bullseye representation of Fig. 5, we notice that P6 and P10 (those who experienced arrhythmogenic episodes) are characterized by a fibrosis distribution confined to a specific region. This probably allowed the signal to propagate across a wide region and, due to the refractoriness heterogeneities, to form loops. On the other hand, for P11 (who did not experience arrhythmia) the fibrotic region is distributed across a large region and at the same time its volume is larger than that of P6 and P10. This heterogeneity might not have created the substrate for a reentry loop, due to the absence of sufficiently long pathways. Notice that these findings (arrhythmogenesis risk related to the substrate heterogeneity rather than its volume) are in agreement with the observations provided in Ref. 20 for the case of acute ischemia.

Another observation in this direction is that all the ectopic beats leading to a reentry (see Fig. 2, bottom) are located, for all three patients, adjacent to segments with full transmural fibrosis (apart from segment 7 in P6).

To conclude, in this study, we have showcased a practical application of a methodology to predict VT formation under BiV-CRT pacing. Our intention was not to conduct extensive statistical analyses, given the relatively modest sample size. Instead, our focus was on presenting a proof-of-concept to highlight the potential of the methodology. The proposed pipeline seems particularly well-suited for systematic application to patient-specific cases, since it requires only standard medical data acquisitions. This methodology could serve as the foundation for a future routine procedure aimed at assessing the arrhythmogenic risk during BiV-CRT pacing, in order to provide useful information to cardiologists on possible treatments (for example, pharmacological or CRT-D).

Despite these encouraging results, some limitations remain:

• *Small patient cohort*: One of the limitations of the study is the small patient cohort ( n=3). While the high predictive consistency is noted, it is statistically insufficient to establish clinical robustness. Moreover, the cohort consists of a specific category of arrhythmic events, that is, iatrogenic arrhythmias occurring post-BiV-CRT implantation. For all these reasons, rigorous validation on a significantly larger, multicenter dataset and with different pathological and therapeutic conditions will be mandatory for future studies in order to make our analysis more robust.
• *Stimulation protocol*: The arrhythmogenic assessment relied on the analysis of only one ectopic beat as trigger of VT formation ( S1–S2 stimulation). Future work should explore the case of a possible train of ectopic beats that originate in a specific location to comprehensively map patient vulnerability.
• *Lack of BiV-CRT EAMS validation*: While the model was personalized against pre-operative EAMS (sinus rhythm) and validated under right pacing conditions (see Ref. 16), the resulting post-operative BiV-CRT activation patterns were not directly validated against EAMS data acquired under biventricular pacing conditions, since these data were not available. Future analyses could focus on filling this gap provided that EAMS will be performed during BiV-CRT.
• *Absence of the His-Purkinje System (HPS)*: The HPS was not explicitly modeled.^40,41^ However, the exclusion of the HPS may lead to an underestimation of the stability and inducibility of reentrant circuits. Literature report suggests that the HPS plays a crucial role in maintaining arrhythmic activity; specifically, propagation through the Purkinje network can prolong reentry by providing additional pathways and allowing earlier activation of distant regions via fast retrograde and anterograde conduction.^19,42^ Therefore, while our results provide a robust indication of arrhythmic propensity, the inclusion of the HPS could further stabilize these loops and potentially increase the predicted risk.
• *Fibrotic ionic model:* We notice that, for the sake of simplicity, the fibrotic action potential has been obtained in this work by performing an ad hoc calibration of the standard TTP06 model, instead of using a fibroblast model as proposed in Ref. 43. However, future developments should incorporate this elaborated ionic model in order to assess the relevance of such models to arrhythmogenesis and the efficacy of our strategy.
• *Absence of the right ventricle*: This work includes only the LV since it is the only chamber where a fibrotic substrate is detected. While the inclusion of the right ventricle would increase anatomical fidelity, we hypothesize that it would not alter the arrhythmogenic outcomes for these specific patients. Indeed, without the presence of further fibrotic heterogeneities, the right ventricle would likely be activated passively without providing the slow-conduction zones necessary to sustain a reentrant loop. Nevertheless, we believe that biventricular simulations will be needed in future studies to better assess the arrhythmogenic risk in CRT patients.

## IV. FINAL REMARKS

Computational methods have been widely used in recent years to study and optimize the BiV-CRT procedure. While the research community has made considerable improvements in this direction, it is still far from providing a tool to be included in the device that could assist clinicians in their decision-making process regarding BiV-CRT. With this work, we contributed in this direction by introducing a multi-objective criterion for personalized BiV-CRT. Our framework highlighted the potential of computational electrophysiology as a clinically relevant tool for risk stratification, noninvasively identifying patients with increased vulnerability to ventricular tachycardia during BiV-CRT pacing. Ultimately, this approach could pave the way for routine computational assessment of the arrhythmic substrate, improving the safety and therapeutic efficacy of treatment for patients undergoing BiV-CRT.

## V. METHODS

### A. Clinical data

Our study includes three BiV-CRT patients, P6, P10, and P11, numbered as in Ref. 44, who were treated at Santa Maria del Carmine Hospital, Rovereto (TN), Italy. These patients suffered from ventricular dyssynchrony caused by Left Bundle Branch Block (LBBB). The etiology of the LV dysfunction was non-ischemic in all three patients. In addition, all patients exhibited a fibrotic region detected by imaging.

For each patient, the available clinical data comprise the following:

• *The device characteristics and the properties of stimulation:* The patients underwent implantation of a BiV-CRT consisting of three electrodes: the first one positioned in the right atrium, the second one at the right ventricle apical endocardium, and the third at the LV epicardium via the coronary sinus. Specifically, the LV lead was targeted to the *Latest Electrically Activated Segment* (LEAS).^45^ For each patient, a specific VV-delay between the electrodes was set, with the right lead delayed with respect to the left one (the values of the delays are reported in Table III).
• *Magnetic Resonance Imaging (MRI) acquisitions:* These data consist of Cine Steady-State Free Precession (Cine-SSFP) short-axis sequences and Phase Sensitive Inversion Recovery (PSIR) sequences with Late Gadolinium Enhancement (LGE), which is a key factor in assessing both ischemic and non-ischemic cardiomyopathies.
• *Electro-Anatomical Mapping System (EAMS) data:* These provide the electrical activation times obtained during sinus rhythm (pre-operative scenario) for all mapped points acquired during the BiV-CRT procedure. Notice that EAMS acquisition is performed by cardiologists during the CRT procedure and no additional invasive mapping is required for our purposes.
• *Follow-up data:* Continuous monitoring via the BiV-CRT device provides detailed registration and documentation of arrhythmic events. The significant clinical follow-up for the three patients is reported in Table III. Based on these documented events, for the purpose of this study, patients P6 and P10 are classified as arrhythmic, whereas patient P11 is classified as non-arrhythmic.

### B. Reconstruction of patient-specific geometries

To define the computational domain for the electrophysiology problem, the initial step involves extracting the LV geometries and the myocardial fibrotic regions from the patient's MRI data. The LV geometry is extracted from Cine-SSFP images thanks to their high resolution, while fibrosis is extracted from LGE-MRI, which clearly delineates the scar shape. To ensure precise anatomical alignment of the functional (Cine-SSFP) and fibrosis (LGE-MRI) planes, the images are synchronized at mid-diastole, which is the conventional acquisition phase for LGE-MRI.

**FIG. 6.**
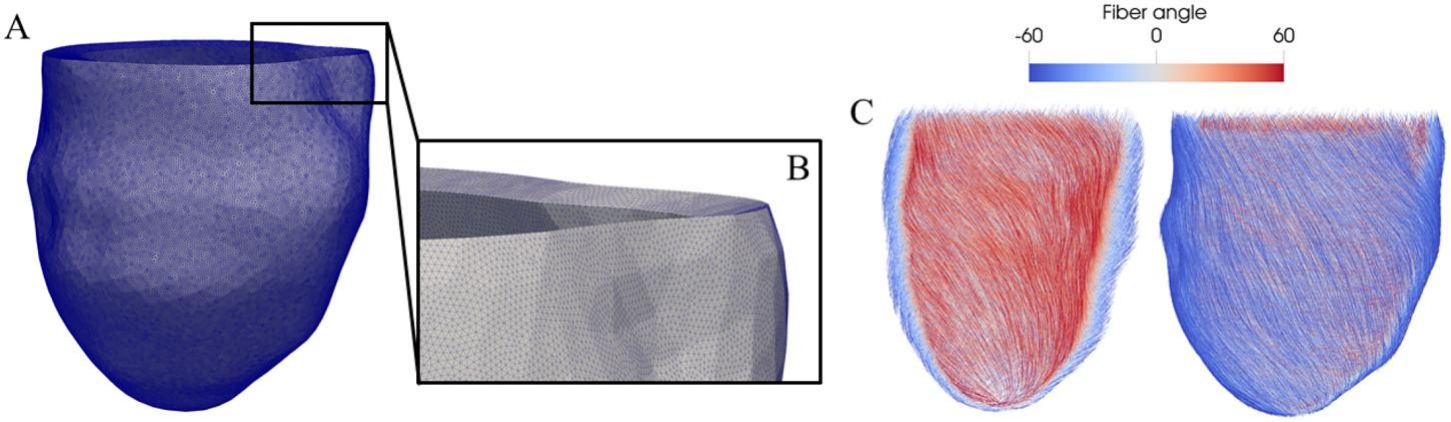
Finite-element mesh and myocardial fiber orientation for patient P6. (a) 3D LV geometry discretized using tetrahedral elements. (b) A zoomed-in view highlighting the resulting tetrahedral mesh. (c) View of the computed myocardial fiber orientation, where the color bar indicates the fiber angle in degrees with respect to the circumferential direction.

More specifically, starting from the MRI images, segmentation of the ventricles is performed using the free, open-source software *3D Slicer.*^46^ Subsequently, *VMTK* (Vascular Modeling Toolkit)^47^ is employed to incorporate the fibrotic tissue into the left ventricle (LV) geometry, completing the 3D reconstruction. Final pre-processing is achieved using *Paraview,*^48^ an open-source data analysis and visualization application, which allows for the spatial identification and differentiation of the two regions: healthy tissue and fibrotic tissue. The resulting reconstructed geometries are shown in Fig. 5, right. In the same figure, on the left, we report a detailed bullseye representation, where for each of the 17 segments we identify the endocardial, myocardial, and epicardial regions, each colored in gray in the presence of fibrosis. This allows us to obtain more specific information about the presence of fibrosis across the transmural depth. Using VMTK, we generate for each patient the finite-element mesh consisting of tetrahedral elements. The average element dimension is set to h=0.7 mm, which used in combination with P2 Finite Elements (see Sec. V C) guarantees the required resolution for accurate electrophysiology simulations. The resulting mesh for a representative patient is shown in Fig. 6.

The complex fiber–sheet architecture that characterizes the myocardium is built using a Laplace–Dirichlet rule-based method,^32^ with input parameters equal to −60° and +60° for epicardial and endocardial helical angles, respectively^32–35^ (see Fig. 6 for a representative patient).

**FIG. 7.**
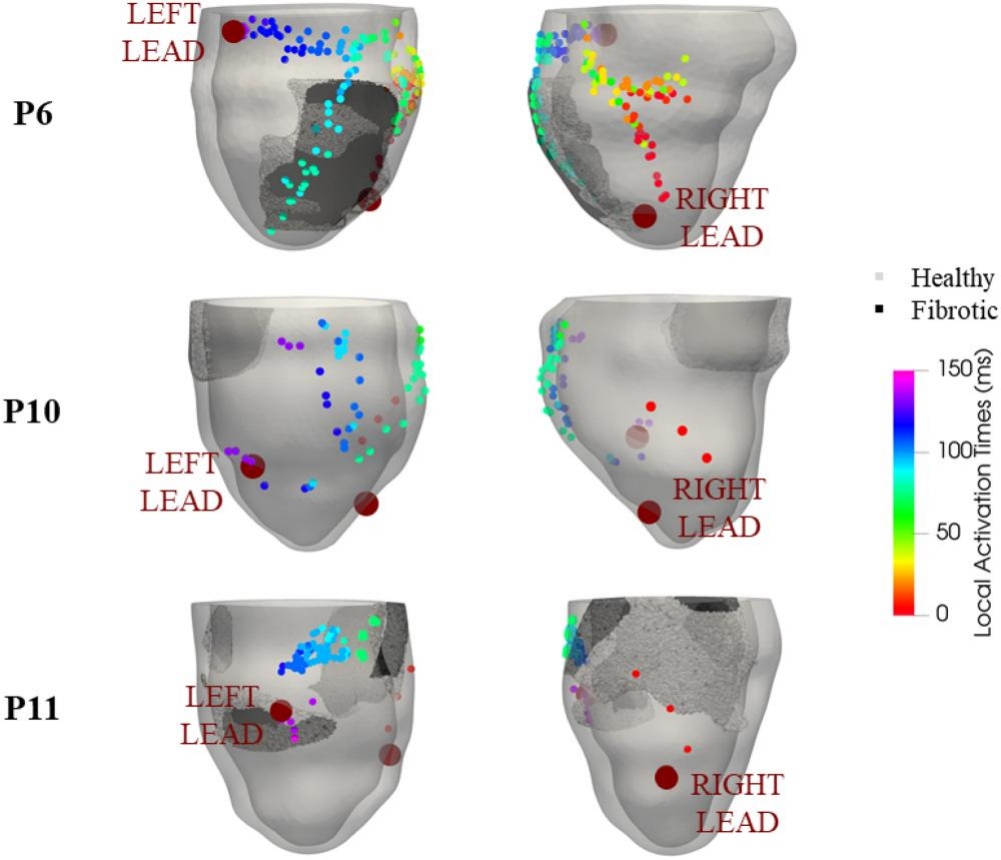
Pre-operative local activation time (LAT) from the EAMS and pacing sites for the three patients. Colored dots show the LAT distribution, while large burgundy spheres mark the pacing electrode locations: the left column shows the left lead placed at LEAS; the right column shows the right lead applied at the interventricular septum.

As regards the computational setup for the clinical BiV-CRT protocol, we follow the methodology described in Ref. 16. Specifically, we neglect the atria and thus consider only the left and right leads. The former is located at LEAS, whereas the latter is located on the LV epicardium. The specified lead locations are visible in Fig. 7.

### C. Overview of the computational model

We consider the personalized computational model proposed for BiV-CRT patients in Ref. 16, applied here to the data and computational meshes of the new cases described in Secs. V A and V B. This model has been validated in Ref. 16, allowing accurate prediction of electrophysiology patterns not used in the personalization procedure.

The model is based on the monodomain equation for the transmembrane potential^49,50^ combined with the Ten Tusscher–Panfilov (TTP06) model for ionic currents,^27^ adapted to the fibrotic case as detailed in Sec. V D (see the supplementary material for the full system of equations). Starting from the activation times obtained from EAMS and according to the calibration procedure introduced in Refs. 24 and 25, we personalize the values of the monodomain conductivities for each patient, differentiating their values in the healthy and fibrotic regions, see Table I. In the same table, we also report estimates of the conduction velocities associated with these conductivities. To find these values, we performed five different measurements of the Euclidean distance between two points on the endocardium in the healthy zone and five in the fibrotic zone, and we divided them by the time the electrical impulse needs to traverse these paths. Subsequently, we calculate the mean value of these estimates.

All the numerical experiments are run using $\text{lif}e^{X}$,^51^ an open-source C++ library for the simulation of heart function, developed at MOX, Dipartimento di Matematica, in collaboration with LaBS, Dipartimento di Chimica, Materiali e Ingegneria Chimica “Giulio Natta” (Politecnico di Milano). See Ref. 52 for details on electrophysiological solvers and the relative open-source binary tool. We use a time discretization parameter equal to $\Delta t=5\times 10^{-5}\;s$^53^ and ${\mathbb{P}}^{2}$ Finite Elements for space discretization, for which the average value of h=0.7 mm in the meshes reported in Sec. V B guarantees sufficient accuracy in approximating the transmembrane potential dynamics. Specifically, it has been proved in previous studies^20,32^ that the accuracy obtained with ${\mathbb{P}}^{2}$/*h* = 0.7 mm is comparable to that obtained with ${\mathbb{P}}^{1}$/*h* = 0.35 mm. In particular, in Ref. 20 we performed a sensitivity analysis that highlighted that the accuracy of ${\mathbb{P}}^{2}$/*h* = 0.7 mm is even of the same order as that of ${\mathbb{P}}^{1}$/*h* = 0.1 mm, thus higher than the accuracy usually required with ${\mathbb{P}}^{1}$.

In Table IV, we report the number of nodes, mesh elements (cells), Degrees of Freedom (DoFs), and the computational cost associated with a simulation (including the ectopic impulse) with a final time of 2.0 s. We run the simulations using 112 parallel processes on the Leonardo supercomputer at the CINECA high-performance computing center (Italy).

**TABLE IV.** Computational details for the three patients. Computational time is reported for a simulation with a final time of 2.0 s.

	P6	P10	P11
No. of nodes	2 262 881	1 218 422	1 606 984
No. of cells	12 849 980	6 691 435	8 995 059
No. of DoFs	17 230 079	9 091 332	12 101 763
CPU	480 min	261 min	360 min

### D. Ionic current model for non-ischemic fibrosis

In this section, we present the mathematical model for the ionic currents: for the healthy region, we consider the standard TTP06 model with standard parameters' values reported in Ref. 27; instead, for the fibrotic region we adapted this model to the case of a fibrotic non-ischemic (and non-hypertrophic) region. To this aim, we refer to Ref. 43, where the authors proposed a fibroblast cell activation model suitably adapting the standard physiological TTP06 model.^27^ Specifically, we refer to Fig. 3(a) in Ref. 43 and to the curve corresponding to a resting potential value of the isolated fibroblast $E_{f}=-39\text{ mV}$ therein. This value is recognized as suitable in the case of pathological fibroblasts, see Refs. 54–58.

Instead of considering a fibroblast cell activation model, here we prefer to maintain the original TTP06 mathematical structure and manually tune its conductances and ion concentrations within the fibrotic regions so as to reproduce the target fibrotic action potential (AP) morphology selected in Ref. 43. The parameters in the adapted TTP06 ionic model are found through a manual iterative tuning process (trial-and-error). The methodology consists of progressively adjusting the conductances and ionic concentrations of the original TTP06 model to match the specific electrophysiological features, such as the resting membrane potential, peak amplitude, and action potential duration, of the target fibrotic AP morphology described by Ref. 43. While an automated sensitivity analysis is not employed, the manual tuning is performed systematically until the simulated AP morphology (shown in Fig. 4) shows agreement with the target reference.

Using this selected curve as a target, we obtained the AP morphology reported in Fig. 4. Notice that this curve and the corresponding one in Ref. 43 represent the average voltage of fibroblasts in the context of the myocardium and not that of an isolated fibroblast.

The resulting tuned AP morphology effectively reproduces the electrical remodeling typical of fibrotic tissue: notice a reduction in the voltage spike, an increment in the resting potential, and a shortening of the AP duration. Specifically, these changes amount to approximately 27.6%, 21.0%, and 19.8%, respectively.

In Table V, we report the values of the adapted parameters in the TTP06 model. The original TTP06 equations affected by these parameter changes are reported in the supplementary material.

**TABLE V.** Values of the parameters used in the adapted TTP06 model. For the other parameters, not reported here, we use for the fibrotic region the same values of the healthy one, as in Ref. 27. ${\left[K^{+}\right]}_{o}$: extracellular concentration of potassium (mmol/L); $G_{kr}$: maximal conductance of potassium current $I_{Kr}(\text{nS}/\text{pF})$; $G_{to}$: maximal conductance of potassium current $I_{to}(\text{nS}/\text{pF})$; $G_{K1}$: maximal conductance of potassium current $I_{K1}(\text{nS}/\text{pF})$; $G_{pK}$: maximal conductance of potassium current $I_{pK}(\text{nS}/\text{pF})$; $G_{Na}$: maximal conductance of sodium current $I_{Na}(\text{nS}/\text{pF})$; $G_{\text{CaL}}$: maximal conductance of calcium current $I_{\text{CaL}}(\text{nS}/\text{pF})$; and $k_{\text{NaCa}}$: maximal scaling coefficient of sodium-calcium exchanger current (pA/pF).

Region	${\left[K^{+}\right]}_{o}$	$G_{Kr}$	$G_{to}$	$G_{K1}$	$G_{pK}$	$G_{Na}$	$G_{\text{CaL}}$	$k_{\text{naca}}$
Healthy	5.4	0.153	0.3	5.4	0.15	14.8	0.000 040	1000
Fibrotic	10.0	0.015	8.0	1.0	0.05	25.0	0.000 006	500

### E. Virtual stimulation procedure

In this work, the stimulation procedure is adapted from established virtual stimulation protocols used in computational studies of VT formation^19,20^ to model the BiV-CRT case.

The protocol for each simulation consists of two sequential steps:

• *S1: BiV-CRT pacing:* The simulation is initiated by applying two electrical impulses corresponding to the left and right ventricular leads. The first impulse is delivered at time t=0 ms, while the second impulse is applied after a time delay specified in Table III. The precise locations of these virtual leads are illustrated in Fig. 7.
• *S2: Ectopic impulse:* Following the BiV-CRT pacing sequence, a single ectopic impulse is applied in the vicinity of the fibrotic region. This action is designed to simulate a pathological trigger arising from cells that exhibit abnormal automaticity or activity triggered by the presence of an anatomical obstacle, such as myocardial fibrosis.^59^

Specifically, for each ectopic impulse, we use the procedure explained in Ref. 20 to search for the *Coupling Interval* (CI), that is, the time period from the first impulse to the first ectopic beat able to depolarize the tissue. Moreover, the stimulus amplitude is set equal to 34.28 V/s, the impulse radius is set to 0.7 mm (independently of the two typologies of impulse described just above), and the impulse duration is set to 3 ms.

To assess the arrhythmogenic risk of each patient, we execute multiple simulations for a set of 10 distinct ectopic sites selected around the fibrotic region. Specifically, the selection is performed on the epicardial surface within the myocardial tissue surrounding the fibrotic region, at a maximum distance of approximately 1 mm. This choice is motivated by the fact that fibroblasts in the fibrotic region can be activated by a propagating action potential but are unable to fire an ectopic impulse themselves through automaticity.^60^ However, fibroblasts have been shown to modulate the ectopic activity of nearby myocytes:^61^ the transitional region where excitable myocytes interact with the fibrotic tissue represents a highly vulnerable zone for ectopic activations.^62^ Notice also that the choice of 10 sites ensures a representative spatial sampling of this area. Compared to a single point, which would offer limited information on the patient's global vulnerability, these 10 sites, positioned to be equally spaced along the fibrotic border, allow us to explore the entire vulnerable substrate while maintaining computational feasibility. The aim of this study is to quantify a patient's vulnerability to arrhythmias by considering the number of ectopic impulses that successfully generate at least one reentry loop. Indeed, the formation of a reentry loop is considered a strong computational signal indicating the possible initiation of reentrant VT.

## SUPPLEMENTARY MATERIAL

See the supplementary material for the complete set of equations for the mathematical models, specifically for the monodomain and the Ten Tusscher-Panfilov model.

## Data Availability

The data that support the findings of this study are available from the corresponding author upon reasonable request.
